# Poisonous Soap

**Published:** 1876-10

**Authors:** 


The New York *Evening Post* of Sept. 5th contained an editorial article entitled "A Perfumed Danger." This is one of many articles we have recently noticed in various newspapers relating to poisonous soaps. It encourages the hope that the people are waking up to the danger they incur by using soaps found almost exclusively in every market. The article says: "Much of the soap which we use is made from fat collected in tenement houses, hospitals and jails, or from the flesh of animals which have died of disease; and there is good evidence of the fact that diseases are sometimes carried in perfumed toilet soap into unsuspecting households. Scarlet fever, especially, is propagated in this way, according to the testimony of physicians." The writer might have added a long list of obstinate skin diseases produced in the same manner. He simply states facts, and suggests a remedy which it seems to us is altogether inadequate to effect a reform. Most of the soap manufactured in this country is made from grease that is purchased from the fat rending establishments at about two-thirds the price of good tallow.

The putrid carcasses of horses, pigs, dogs, and all animals from which fat can be extracted, find a ready market at these places.

The soap maker puts this grease into large open kettles, in the bottom of which are steam coils for boiling the material used. A lye made from caustic soda, of just sufficient strength to combine with the grease and make what is called a "neutral soap," is added. The contents of the kettle is boiled nearly a week, and during the time much of the weak lye is drawn off and stronger lye added. There are certain things that indicate to the soap maker the completion of the process, when the soap is put into frames to harden, and is then cut, boxed, and marketed. To make toilet soaps this soap is melted down, scented, and pressed into cakes.

Now, every bar and cake of such soap should be marked "dangerous." It is about as safe to eat under-cooked, lean pork, as to use such soaps, and for reasons that any competent chemist will readily understand. When grease and alkali are *completely* combined, that is when there is *perfect* saponification, a new substance is formed called soap, which no chemist can again separate. It is no longer grease and alkali, but soap. So long as the combination is perfect, it matters not how impure the materials used may have been, its poisonous properties are destroyed. But complete saponification is not possible at the low temperature of the open kettle process. If the alkali is strong enough to saponify the grease, it renders the soap unfit for use; it is, therefore, used too weak to saponify *all* the grease, and a portion of it—estimated at about one-fifth—remains in the soap in an unchanged condition. It is this that imparts the carrion odor to all common soaps, when not covered by perfumery. These soaps scatter the seeds of disease broadcast throughout the land.

The evil will never cease until public opinion and the want of patronage compel the soap makers to abandon the open kettle process. The best soaps are made in strong close boilers, in which are agitators to thoroughly mix the materials during the process. The soap is made at a very high temperature, and at a pressure of one hundred and eighty pounds to the square inch, at which point perfect saponification takes place at once, and the soap comes from the boiler clean and odorless, even when made from rancid grease. A beautiful soap is in this way made from cotton-seed oil, which it is impossible to make in the open kettle. Owing to the saponification of *all* the grease and saving of the glycerine, which is entirely lost in the open kettle process, the yield of soap is much greater, and its quality far superior to any other. The cost of fitting up a factory for making soaps by this process is not more than half that of a factory of the same capacity where open kettles are used; and it requires but little skill and experience to run it successfully. With all these advantages, it is surprising that there are less than a dozen such factories in the United States. There should be one in every city. We have used no other soap for ten years, and could not be induced to use any other.

